# Supplementary material for: HER2 Status in High-Risk Endometrial Cancers (PORTEC-3): Relationship with Histotype, Molecular Classification, and Clinical Outcomes
Source: Cancers (Basel). 2020 Dec 25;13(1):44. doi: 10.3390/cancers13010044 (PMC7795222; doi:10.3390/cancers13010044)
Supplement: Supplementary file 1 [file cancers-13-00044-s001.pdf]

Supplementary Materials: HER2 Status in High-Risk Endometrial Cancers (PORTEC-3): Relationship with Histotype, Molecular Classification, and Clinical Outcomes

Lisa Vermij, Nanda Horeweg, Alicia Leon-Castillo, Tessa A. Rutten, Linda R. Mileshkin, Helen J. Mackay, Alexandra Leary, Melanie E. Powell, Naveena Singh, Emma J. Crosbie, Vincent T.H.B.M. Smit, Carien L. Creutzberg and Tjalling Bosse

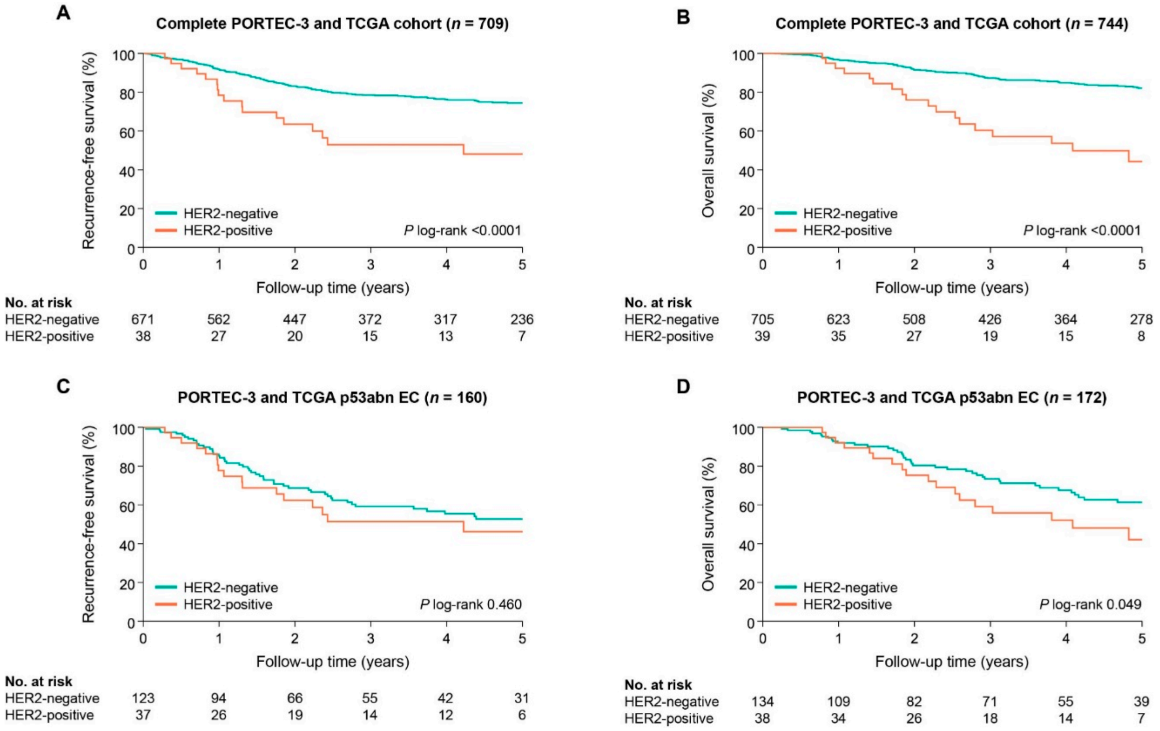

Figure S1. Kaplan–Meier survival curves for (A) recurrence-free survival (RFS) and (B) overall survival (OS) in patients with HER2-positive and HER2-negative endometrial cancer (EC) from the combined PORTEC-3 and TCGA UCEC cohorts; (C) RFS and (D) OS for patients with HER2-positive and HER2-negative p53-abnormal EC.

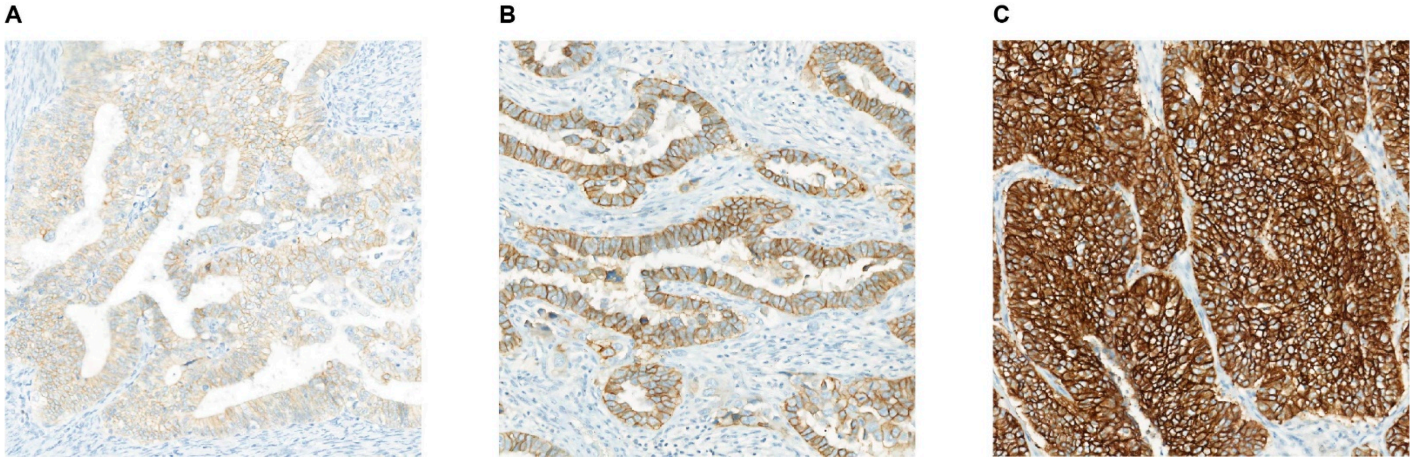

Figure S2. Representative examples of faint, moderate and strong membranous HER2 immunoreactivity in endometrial cancer. Representative examples of HER2 immunohistochemistry with (**A**) faint membranous immunoreactivity, (**B**) moderate membranous immunoreactivity, and (**C**) strong membranous immunoreactivity.

Table S1. Multivariable analysis of HER2 status and clinicopathological features in the combined PORTEC-3 and TCGA Uterine Corpus Endometrial Carcinoma cohort.

	**Recurrence–Free Survival 164 Events**				**Overall Survival 121 Events**			
	Total *n*	HR	95% CI	*p*–value	Total *n*	HR	95% CI	*p*–value
Age	684	1.009	0.990–1.028	0.38	717	1.033	1.009–1.058	0.006
HER2 status								
Negative	647	1			679	1		
Positive	37	1.180	0.670–2.077	0.57	38	1.389	0.784–2.460	0.26
Molecular subgroups								
MMRd	224	1			237	1		
p53abn	152	2.291	1.447–3.628	<0.0001	163	2.045	1.230–3.398	0.006
*POLE*mut	78	0.055	0.008–0.398	0.004	80	0.239	0.073–0.786	0.018
NSMP	230	0.943	0.620–1.435	0.79	237	0.685	0.391–1.197	0.18
Histology and grade								
Endometrioid, low grade	318	1			327	1		
Endometrioid, high grade	188	1.156	0.745–1.794	0.52	203	1.821	1.068–3.104	0.028
Non–endometrioid	178	0.869	0.531–1.423	0.58	187	1.325	0.736–2.386	0.35

Stage								
I–II	442	1			458	1		
III–IV	242	2.146	1.552–2.968	<0.0001	259	2.200	1.512–3.202	<0.0001
LVSI								
Absent	326	1			340	1		
Present	358	1.270	0.903–1.787	0.17	377	1.544	1.019–2.341	0.040

Abbreviations: HR, hazard ratio; POLEmut, POLE (ultra-)mutated; MMRd, mismatch repair deficient; NSMP, No Specific Molecular Profile; p53abn, p53 abnormal; LVSI, lymphovascular space invasion.

Table S2. Comparison of HER2 status assessed by immunohistochemistry, scored by adjusted ASCO/CAP 2007 breast cancer guideline for HER2 testing, and dual in situ hybridization.

**Adjusted ASCO/CAP 2007 BC Guideline***	**HER2 DISH**				
	**Not Amplified (*n*)**	**Amplified (*n*)**	**Failed (*n*)**	**Not Analysed (*n*)**	**Total (*n*)**
IHC 0	10	0	0	253	263
IHC 1+	17	2	2	34	55
IHC 2+	44	6	1	1	52
IHC 3+	6	16	0	0	22
Failed	13	0	0	0	13
Total	90	24	3	288	405

Abbreviations: BC, breast cancer; DISH, dual in situ hybridisation.

***Adjusted ASCO/CAP 2007 breast cancer scoring criteria.**

	**Original Scoring Criteria**	**Adjusted Scoring Criteria**
IHC 0	No staining observed	No staining observed

IHC 1+	Weak, incomplete membrane staining in any proportion or weak, complete membrane staining in <10% of cells	Weak, incomplete membrane staining in any proportion or weak, complete membrane staining in <10% of tumour cells or moderate (in)complete membrane staining in <10% of tumour cells
IHC 2+	Complete membrane staining that is nonuniform or weak but with obvious circumferential distribution in >10% of cells, or intense complete membrane staining in ≤30% of tumour cells	Weak, complete or moderate, (in)complete membrane staining in >10% of tumour cells or intense, (in)complete membrane staining in ≤30% of tumour cells
IHC 3+	Uniform intense membrane staining in >30% of tumour cells	Intense, (in)complete membrane staining in >30% of tumour cells
